# *OsTCTP*, encoding a translationally controlled tumor protein, plays an important role in mercury tolerance in rice

**DOI:** 10.1186/s12870-015-0500-y

**Published:** 2015-05-20

**Authors:** Zhan Qi Wang, Ge Zi Li, Qiao Qiao Gong, Gui Xin Li, Shao Jian Zheng

**Affiliations:** State Key Laboratory of Plant Physiology and Biochemistry, College of Life Sciences, Zhejiang University, Hangzhou, 310058 China; College of Agronomy and Biotechnology, Zhejiang University, Hangzhou, 310058 China

**Keywords:** *Oryza sativa* L, Mercury stress, Antioxidant enzyme, Hydrogen peroxide levels, N-glycosylation

## Abstract

**Background:**

Mercury (Hg) is not only a threat to public health but also a growth risk factor to plants, as it is readily accumulated by higher plants. Accumulation of Hg in plants disrupts many cellular-level functions and inhibits growth and development; however, the detoxification and tolerance mechanisms of plants to Hg stress are still not fully understood. Exposure to toxic Hg also occurs in some crops cultivated under anoxic conditions, such as rice (*Oryza sativa* L.), a model organism and one of the most important cultivated plants worldwide. In this study, we functionally characterized a rice *translationally controlled tumor protein* gene (Os11g43900, *OsTCTP*) involved in Hg stress tolerance.

**Results:**

*OsTCTP* was ubiquitously expressed in all examined plant tissues, especially in actively dividing and differentiating tissues, such as roots and nodes. OsTCTP was found to localize both the cytosol and the nucleus. OsTCTP was induced by mercuric chloride, cupric sulfate, abscisic acid, and hydrogen peroxide at the protein level in a time-dependent manner. Overexpression of *OsTCTP* potentiated the activities of several antioxidant enzymes, reduced the Hg-induced H_2_O_2_ levels, and promoted Hg tolerance in rice, whereas knockdown of *OsTCTP* produced opposite effects. And overexpression of *OsTCTP* did not prevent Hg absorption and accumulation in rice. We also demonstrated that Asn 48 and Asn 97 of OsTCTP amino acids were not the potential N-glycosylation sites.

**Conclusions:**

Our results suggest that OsTCTP is capable of decreasing the Hg-induced reactive oxygen species (ROS), therefore, reducing the damage of ROS and enhancing the tolerance of rice plants to Hg stress. Thus, *OsTCTP* is a valuable gene for genetic engineering to improve rice performance under Hg contaminated paddy soils.

**Electronic supplementary material:**

The online version of this article (doi:10.1186/s12870-015-0500-y) contains supplementary material, which is available to authorized users.

## Background

Mercury (Hg) is deadly toxic to humans and ecosystems, which is considered as a global pollutant because it is highly mobile and extremely persistent in the environment [1]. It is not only a threat to public health [2] but also a growth risk factor to plants [3], as it is readily accumulated by higher plants [4-6]. As a phytotoxic metal, Hg has wide-ranging adverse effects on the physiological activities of plants, such as prevention of water and mineral nutrient uptake [7,8] and inhibition of photosynthesis [9,10]. Furthermore, mercuric ions react specifically with sulfhydryl groups of proteins and other biomolecules and induce the Fenton reaction, resulting in oxidative stress, lipid peroxidation [11-13], cell damage and signaling disruption in plants [3,14]. Exposure to toxic Hg also occurs in some crops cultivated under anoxic conditions, such as rice [15,16]. Recent studies have demonstrated that rice intake is the major pathway for human exposure to Hg in inland China [17,18]. So, it is of immense importance to elucidate the detoxification and tolerance mechanisms of rice plants to Hg and consequently optimize rice performance under Hg contaminated paddy soils.

The translationally controlled tumor protein (TCTP), also variously known as Q23 [19], P21 [20], P23 [21], IgE-dependent histamine-releasing factor (HRF) [22], and fortilin [23], is ubiquitously found in all eukaryotes. As implicated in its name, TCTP is regulated at both translational and posttranslational levels in response to a wide range of extracellular signals and conditions and exerts diverse functions in various cellular processes such as cell growth, proliferation, cell cycle progression, DNA repair, malignant transformation, anti-apoptosis as well as protection against various cellular stresses and other immunological functions [24-26]. In *Arabidopsis*, TCTP has been shown to be an important growth regulator and expressed throughout plant tissues and developmental stages with increased expression in meristematic and expanding cells [27]. Knockdown of *AtTCTP* by RNA interference slows vegetative growth and exhibits severe dwarf phenotype due to a reduction of cell numbers, whereas homologous *tctp* plants (T-DNA insertion lines) are embryonic lethal [25]. Overexpression of *AtTCTP* enhances drought tolerance with rapid ABA-induced stomatal closure [28]. Moreover, plant TCTP protein is also proposed to have a role in long-distance movement of phloem proteins and in regulating the hypersensitive response [29,30]. These published results reveal that TCTP not only regulates organismal growth but also asserts plant specific functions.

Previously, through a 2-D gel analysis, OsTCTP was presumed to participate in the Hg tolerance of rice at the protein level [31]. However, the functional analysis of *OsTCTP* in rice is still very scanty, and the molecular and physiological mechanisms of *OsTCTP* in rice Hg tolerance are still not fully understood. Here, we reported the isolation and functional characterization of *OsTCTP* (Os11g43900), which was induced under Hg stress condition in rice [31]. The *OsTCTP* is shown to be more highly expressed in physiologically active and proliferating tissues as would be expected for a component of the target of rapamycin (TOR) pathway. Analysis of a T-DNA insertion line in the promoter region demonstrated that transcriptional knockdown of *OsTCTP* caused compensation in protein abundance due to the translational regulation. Overexpression of *OsTCTP* potentiated the activities of several antioxidant enzymes, whereas knockdown of *OsTCTP* aborted the antioxidant system and increased the Hg-induced H_2_O_2_. Our results suggested that OsTCTP confers tolerance to Hg stress possibly through the regulation of ROS and their scavengers.

## Results

### OsTCTP is a single-copy gene with two differentially spliced transcripts but only one transcript can be translated

**Figure 1:**
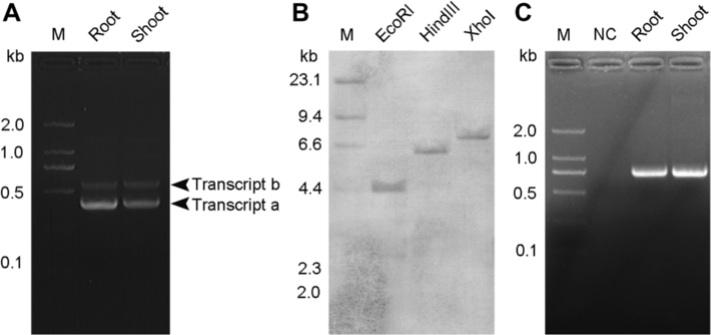
Genomic organization and alternative splicing of *OsTCTP*. **(A)** RT-PCR analysis using a primer pair covering the differentiated regions of the *OsTCTPa* and *OsTCTPb* cDNAs. The root and shoot mRNAs from cv. ‘Nipponbare’ were used for RT-PCR. **(B)** Southern blot analysis of the *OsTCTP* gene. Total DNA from cv. ‘Nipponbare’ (30 μg for each lane) was digested individually with *Eco*RI, *Hin*dIII and *Xho*I and hybridized with a gene-specific probe covering the region from nucleotide 513 to the 3’ end of the *OsTCTPa* cDNA. **(C)** Genomic PCR analysis using two primers covering the differentiated regions of the *OsTCTPa* and *OsTCTPb*. The leaf DNA from cv. ‘Nipponbare’ was used for genomic PCR. M, DNA size markers.

Reverse transcription-polymerase chain reaction (RT-PCR) with a pair of primers spanning an exon junction of *OsTCTP* gene amplified two cDNA fragments (*OsTCTPa* and *OsTCTPb*) from the rice root and shoot RNA samples (Figure 1A). To determine if these two fragments were derived from alternative splicing of a single gene, southern blot hybridization was performed with a probe covering an identical region of both *OsTCTPa* and *OsTCTPb*. One strongly hybridizing band was detected in each of the three digestions (*Eco*RI, *Hin*dIII and *Xho*I) of rice genomic DNA (Figure 1B). Genomic PCR with a pair of primers covering the differentiated region also gave rise to a single band (Figure 1C). Therefore, *OsTCTPa* and *OsTCTPb* most likely resulted from the alternative splicing of a single *OsTCTP* gene in rice plants. Sequence alignments showed that *OsTCTPb* was 190 bp longer than *OsTCTPa* and encoded a putative truncated protein due to an open reading frame shift (Additional file 1: Figure S1). Interestingly, in both root and shoot tissues, *OsTCTPa* was the predominant isoform of *OsTCTP* transcripts. This is consistent with the previous report showing that there are usually several different cDNAs in many plant species [27].

**Figure 2:**
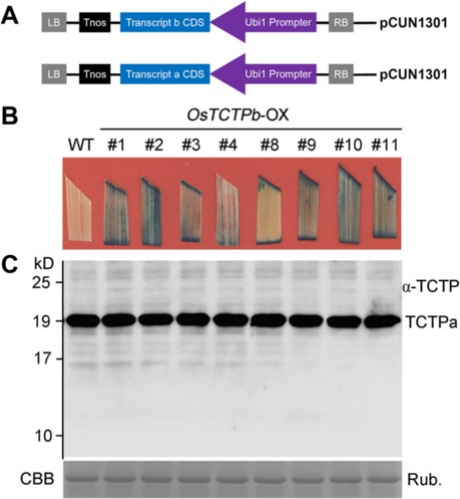
*OsTCTP* expression patterns in transgenic rice plants. **(A)** Structure of constructs for rice transformation. The overexpression constructs (*OsTCTPa* and *OsTCTPb*) were developed under the control of the Ubi1 promoter and nopaline synthase (Nos) terminator cassette. **(B)** GUS staining of *OsTCTPb* overexpression transgenic rice plants. **(C)** Western blot analysis of OsTCTP protein from transgenic plants using an antibody against rice OsTCTP. Equal amounts (20 μg) were loaded to each lane and confirmed by coomassie brilliant blue (CBB) staining of Rubisco (bottom). The experiments were repeated for three times with similar results.

To determine if these two transcripts could be translated into mature proteins, CDSs of both *OsTCTPa* and *OsTCTPb* were subcloned into a binary expression vector pCUN1301, respectively, which were driven by a constructive maize ubiquitin 1 promoter (Figure 2A). Transgenic plants were generated by introducing the constructs into the japonica rice cultivar ‘Nippobare’ by *Agrobacterium tumefaciens*-mediated transformation [32]. Thirty-two independent *OsTCTPa*-OX transgenic plants and 11 independent *OsTCTPb*-OX transgenic plants were generated (T0 generation), each containing one to several copies of the transgene, as confirmed by southern blot analysis (Additional file 1: Figures S2B and S3). We then tested GUS expression in *OsTCTPb*-OX T0 plants and eight of them showed the GUS activity (Figure 2B). The eight *OsTCTPb*-OX T0 plants showing GUS activity were then subjected to western blot analysis to investigate the accumulation of the OsTCTP protein. This revealed that no OsTCTPb protein was detected in neither wild-type (WT) under the control of the native promoter nor the transgenic plants under the control of the constructive promoter (Figure 2C). Taken together, these results strongly indicated that *OsTCTP* is a single-copy gene with two differentially spliced transcripts but only *OsTCTPa* transcript can be translated into mature protein. So, in the following text, OsTCTP refers to the translational product of *OsTCTPa* gene.

### OsTCTP is a typical TCTP protein

**Figure 3:**
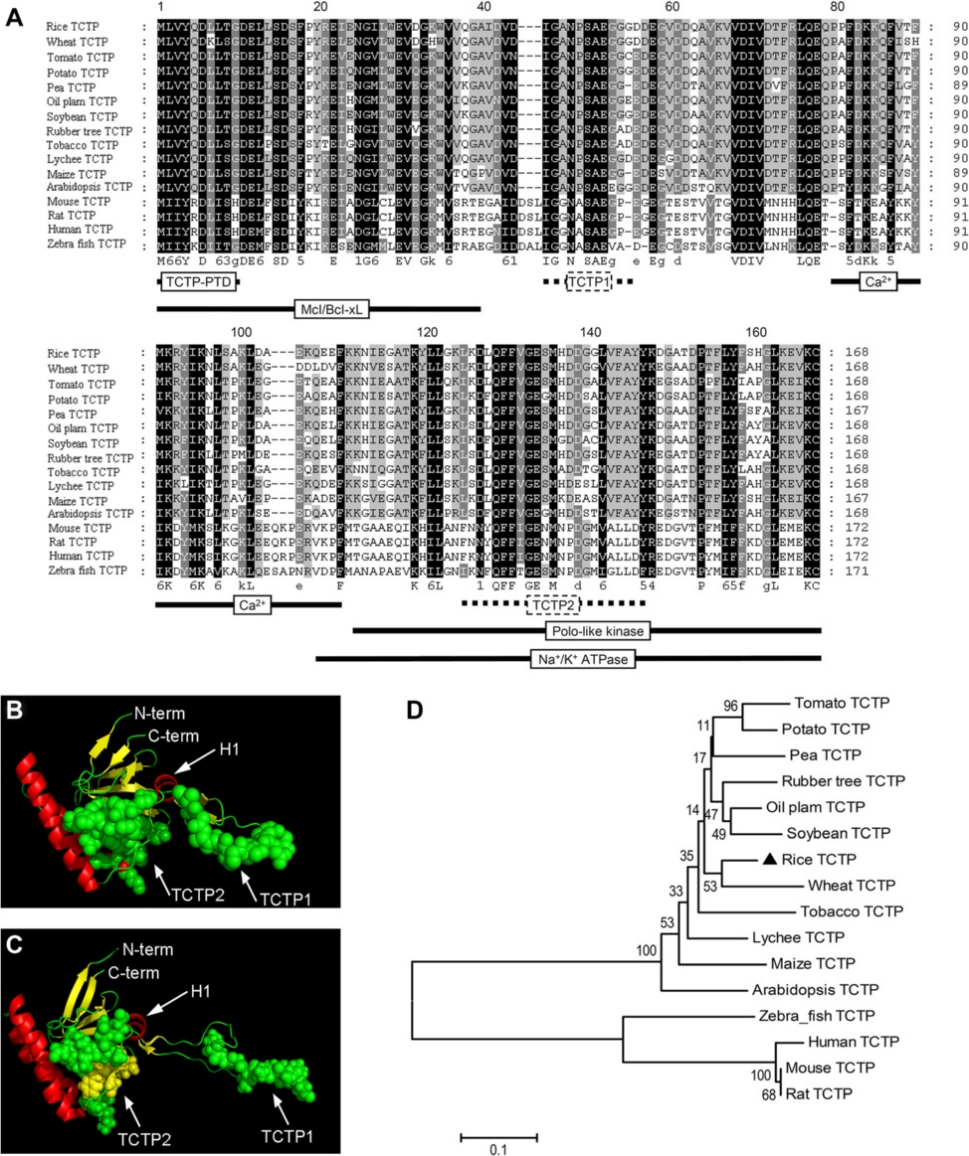
Sequence comparison, structural modeling and phylogenetic analysis of TCTP. **(A)** The amino acid sequence alignment was performed using the ClustalW2 software on representative plant and nonplant sequences. Positions with strictly conserved amino acids are highlighted in black, conserved substitutions in dark gray, and blocks of similar residues in light gray. Domains identified for nonplant TCTPs (Mcl/Bcl-xL interaction [42], Polo kinase interaction [43], Na^+^/K^+^ ATPase interaction [44], Ca^2+^ binding [45], TCTP self-interaction [46] and protein transduction domain [47]) are indicated by black lines and the TCTP signature by a dotted line. **(B)** The putative 3D structure of the OsTCTP. The structure of the OsTCTP protein was modeled using the known structure of the human TCTP (PDB ID 2HR9, http://www.rcsb.org) as a template on the Swiss-Model server (http://swissmodel.expasy.org [73]). The model obtained for the rice protein (b) shows high similarity when compared with the structure of the human protein **(C)**. The conserved domains TCTP1 and TCTP2 of TCTP proteins are shown as calottes. The conserved helix (H1) is marked by an arrow. Helices, β-sheets, and coil regions of the two structures are represented in red, yellow, and green, respectively. **(D)** Phylogenetic analysis of OsTCTP and its 15 close homologs. The numbers under the branches refer to the bootstrap value of the neighbor-joining phylogenetic tree. The length of the branches is proportional to the amino acid variation rates. The scale bar indicates the number of amino acid substitutions per site.

Searching of protein databases for sequences similar to well-characterized TCTPs (human, *Drosophila* and yeast) reveals TCTP homologs in a large number of plant species. Protein sequence comparison of plant and non-plant TCTPs showed amino acid identities in the range of 40% and similarities of ~60% using the Point Accepted Mutation (PAM) matrix [33]. InterProScan [34] analysis showed that OsTCTP has two typical TCTP domains TCTP1 (45–55) and TCTP2 (125–147) (Figure 3A). This comparative analysis indicated that OsTCTP is a TCTP protein at the primary structure of protein. In parallel, we used homology modeling to obtain a 3D-structure prediction for the OsTCTP protein to investigate the possibility that it might have a similar function as TCTP in other organisms. This model showed high similarity to the known structure of the human TCTP protein with a nearly identical spatial organization of α-helices, β-sheets, and an unstructured loop (Figure 3B,C). Furthermore, phylogenetic analysis between OsTCTP and its 15 homologs indicated that OsTCTP is closely related to the wheat TCTP protein (Figure 3D). Based on the data presented in the phylogenetic tree, the TCTP family was divided into two groups. Those from plants such as *Arabidopsis thaliana*, maize (*Zea mays*) and wheat (*Triticum aestivum*) form one cluster, and those from non-plants form the other cluster. The OsTCTP is expectedly mapped to the plants cluster. This result indicated that the phylogenetic clustering of TCTP sequences is almost consistent with the accepted phylogeny of eukaryotes. From these results, it is clear that the OsTCTP is a typical TCTP at both protein and evolution levels.

### OsTCTP gene is regulated at the translational level

**Figure 4:**
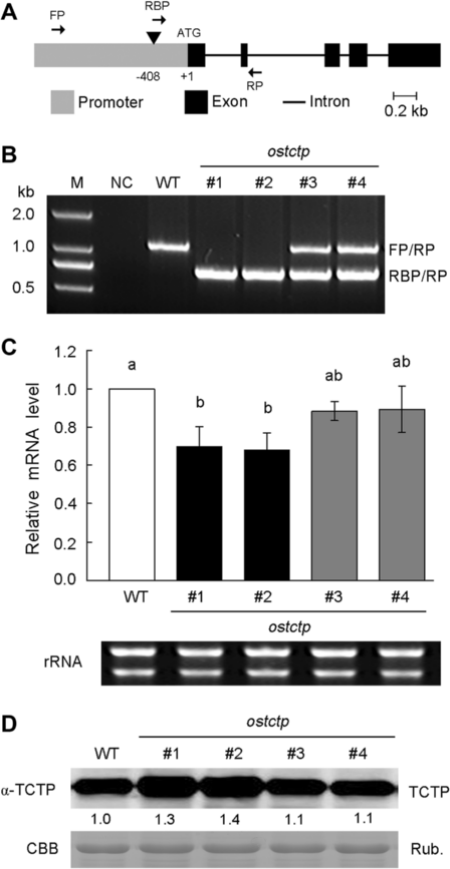
OsTCTP expression patterns in *ostctp* mutant plants. **(A)** Gene structure of *OsTCTP*. Gray box is the promoter region; Block boxes indicate exons in coding region; lines connecting boxes are introns. Triangles are T-DNA insertions of *ostctp*. Arrows FP, RP, and RBP are primers used for genotyping *ostctp*. **(B)** PCR analysis of genotyping of mutant *ostctp*. FP, RP, and RBP are primers used for genotyping *ostctp*. **(C)** qRT-PCR analysis of *OsTCTP* mRNA level in mutant *ostctp. Histone H3* was used as an internal standard and data are given as means ± SD of three biological replicates. Means with different letters are significantly different (P < 0.05, Tukey’s test). **(D)** Western blot analysis of OsTCTP protein from mutant *ostctp* using an antibody against rice OsTCTP. Equal amounts (20 μg) were loaded to each lane and were confirmed by coomassie brilliant blue (CBB) staining of Rubisco (bottom). The experiments were repeated for three times with similar results. Quantitative assessment was processed by Quantity One® Software (http://www.bio-rad.com).

It has been shown that *TCTP* genes are regulated at the translational level in animals [24,35]. To determine whether the synthesis of OsTCTP protein is also regulated at the translational level in rice, we examined the expression patterns of *OsTCTP* by mutant analysis. A mutant line of *OsTCTP* in the background of Dongjin, *ostctp*, with a T-DNA inserted in the promoter region (Figure 4A), was obtained from the Rice T-DNA Insertion Sequence Database (http://cbi.khu.ac.kr) [36]. After being confirmed for the T-DNA insertion site as described previously [37], homozygous and heterozygous mutant plants (Figure 4B) were used for further analysis. Firstly, we examined the mRNA levels in the *ostctp* by qRT-PCR analysis. Notably, compared to the wild-type plants, it was down by approximately 10% to 30% in heterozygous (#3 and #4) and homozygous (#1 and #2) mutant plants, respectively (Figure 4C), indicating that the expression of *OsTCTP* was interrupted at the transcriptional level in the mutant plants. And then, we checked the OsTCTP protein level in wild-type and mutant rice plants using the western blot. However, the data showed that the accumulation of OsTCTP protein was higher in mutant plants than that in the wild-type rice plants, especially in the homozygous mutant plants (#1 and #2, Figure 4D). These results indicated that *OsTCTP* gene is regulated at translational level in rice.

### Expression pattern and subcellular localization of OsTCTP

**Figure 5:**
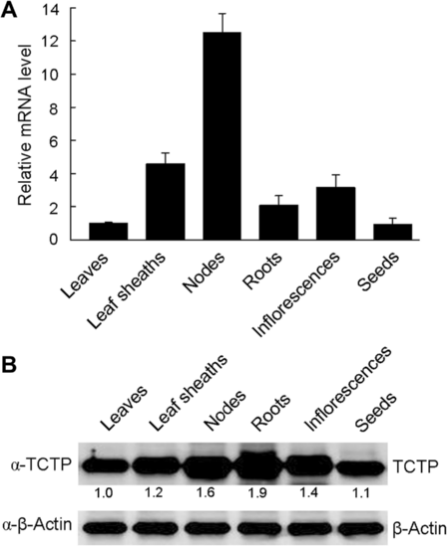
qRT-PCR and western blot analyses of OsTCTP in wild-type rice plants. **(A)** qRT-PCR analysis of *OsTCTP* mRNA level in wild-type rice plants. *HistoneH3* was used as an internal standard and data are given as means ± SD of three biological replicates. Means with different letters are significantly different (P < 0.05, Tukey’s test). **(B)** Western blot analysis of OsTCTP protein from wild-type rice plants using an antibody against rice OsTCTP. Equal amounts (20 μg) were loaded to each lane and β-actin was used as an internal standard. The experiments were repeated for three times with similar results. Quantitative assessment was processed by Quantity One® Software (http://www.bio-rad.com).

To gain insight into TCTP function in rice plants, we investigated its expression pattern in a range of tissues by qRT-PCR and western blot analyses. qRT-PCR analysis revealed that *OsTCTP* was expressed in all analyzed tissues (leaves, leaf sheaths, nodes, roots, inflorescences, and seeds) and predominantly expressed in leaf sheaths, nodes, roots and inflorescences (Figure 5A). Consistent with the results of qRT-PCR analysis, strong immunoblot signals were observed in leaf sheaths, nodes, roots, and inflorescences (Figure 5B), suggesting that *OsTCTP* is ubiquitously expressed in all examined plant tissues, especially in actively dividing and differentiating tissue types, such as roots and nodes.

**Figure 6:**
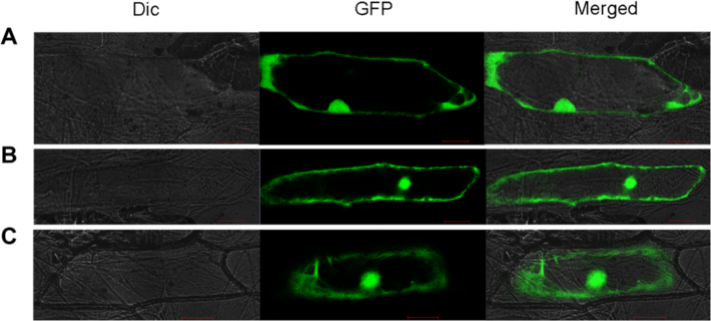
Subcellular localization of OsTCTP. **(A)** GFP protein localization in onion epidermal cells. **(B)** OsTCTP-GFP fusion protein localization in onion epidermal cells. **(C)** Plasmolysed cell transformed with OsTCTP-GFP fusion protein. Dic, bright field images; GFP, GFP fluorescence; and Merged, merged image. Scale bars =100 μm.

To gain insight into further function of OsTCTP in rice plants, we determined its subcellular localization by translationally fusing full-length *OsTCTP* to the N-terminal of the green fluorescent protein (GFP) and expressing the chimeric protein under the control of the CaM35S promoter. Onion (*Allium cepa*) epidermal cells transiently expressing the GFP without OsTCTP showed signals throughout cells (Figure 6A). The fluorescence signal from OsTCTP-GFP fusion protein was observed both in the cytosol and nucleus (Figure 6B), and in the plasmolysed cells, the fluorescence of OsTCTP-GFP was still observed both in the cytosol and the nucleus (Figure 6C). These results suggested that OsTCTP has dual localization and is consistent with its multi-functions in different biochemical reactions.

### Induction of OsTCTP protein accumulation by Hg, Cu, ABA, and H_2_O_2_ in rice

**Figure 7:**
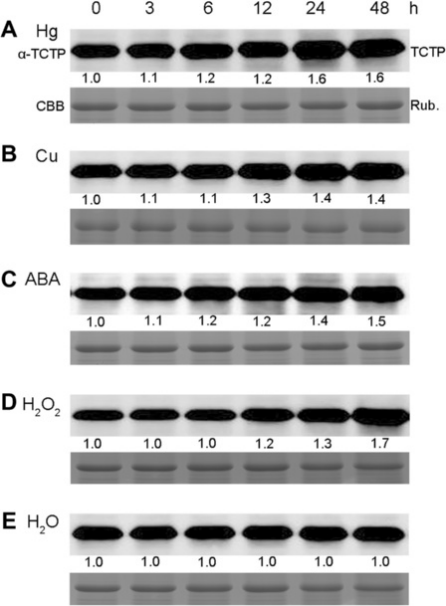
Protein accumulation profiles of OsTCTP under Hg, Cu, ABA and H_2_O_2_ treatments. Western blot analysis of OsTCTP accumulation under different stress conditions. Hydroponically grown 2-week-old rice plants were transferred to solutions containing 25 μM Hg **(A)**, 50 μM Cu **(B)**, 50 μM ABA **(C)**, 100 mM H_2_O_2_ **(D)**, or water (H_2_O, **E)** for the time periods indicated. Equal amounts (20 μg) were loaded to each lane and were confirmed by coomassie brilliant blue (CBB) staining of Rubisco (bottom). Numbers under lanes in **(A–E)** indicate relative band intensities that were quantified and normalized the controls (0 hour) for each panel. Quantitative assessment was processed by Quantity One® Software (http://www.bio-rad.com). The experiments were repeated for three times with similar results.

Previous reports have shown that TCTPs have diverse roles in response to various stresses and some plant hormones [24,27,35]. To examine the role of OsTCTP, we investigated its time-dependent accumulation patterns under different stresses by western blot analysis. As shown in Figure 7, the OsTCTP protein accumulated in seedlings within 3 hours following Hg, Cu, and ABA treatments (Figure 7A–C). In contrast, the accumulation of OsTCTP protein arosed after 12 hours and then increased strongly over 48 hours of H_2_O_2_ stress treatment (Figure 7D). There was no visible accumulation of OsTCTP protein in seedlings treated with H_2_O only (Figure 7E). The protein level of OsTCTP was slightly accumulated under the NaCl stress and mechanical wound (Additional file 1: Figure S4A,B). However, the OsTCTP accumulation profile was not affected by PEG, SA, JA, and NAA treatments (Additional file 1: Figure S4C–E). These results revealed that OsTCTP is likely involved in some abiotic stress responses in rice plants.

### Phenotypic analysis of OsTCTP-OX and OsTCTP-RNAi transgenic plants

To analyze the function of *OsTCTP* in rice, we constitutively increased or suppressed the expression of *OsTCTP* in transgenic rice plants. The transgenic lines were generated by introducing the overexpression construct (*OsTCTP*-OX) or the double-stranded RNA interference construct (*OsTCTP*-RNAi) into wild type of cv. ‘Nipponbare’. Thirty-two independent overexpression lines were generated using the *OsTCTP*-OX construct. Western blot analysis indicated that 28 lines showed high constitutive expression in the *OsTCTP*-OX transgenic plants (Additional file 1: Figure S2A) and 22 of them were selected to analyze by southern blot and 5 *OsTCTP*-OX lines contained a single-copy insertion (Additional file 1: Figure S2B). Based on the above analyses, two lines (*OsTCTP*-OX-#12 and *OsTCTP*-OX-#14) were selected for further phenotypic and functional characterization. In addition, twenty-six independent suppression lines were generated using the *OsTCTP*-RNAi construct. Western blot analysis indicated that 20 lines showed low expression in the *OsTCTP*-RNAi transgenic plants (Additional file 1: Figure S5A) and 22 of them were selected to analyze by southern blot and 5 *OsTCTP*-RNAi lines contained a single-copy insertion (Additional file 1: Figure S5B). The production of endogenous OsTCTP protein was blocked almost completely and moderately in *OsTCTP*-RNAi-#10 and *OsTCTP*-RNAi-#17, respectively and they were selected for further functional analysis.

**Figure 8:**
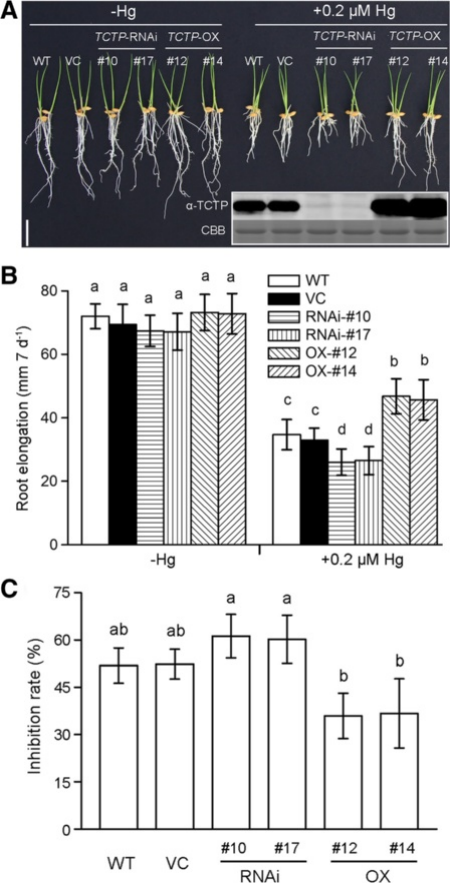
Phenotypic analysis of the wild type (WT), vector control (VC), *OsTCTP*-OX and *OsTCTP*-RNAi transgenic rice plants. **(A)** Hg tolerance of WT (cv. Nipponbare), VC (vector control), *OsTCTP*-RNAi (#10 and #17) and *OsTCTP*-OX (#12 and #14). Germinated seeds were exposed to 0.5 mM CaCl_2_ solution (pH 5.5) containing 0 or 0.2 μM HgCl_2_ for 7 days. Scale bar = 2 cm. **(B)** Root length of WT (cv. Nipponbare), VC (vector control), *OsTCTP*-RNAi (#10 and #17) and *OsTCTP*-OX (#12 and #14). Root length was measured before and after the treatment. **(C)** The inhibition rate was defined as [1 – (the ratio of the root elongated of the plants receiving Hg treatment to that of the no-Hg control) × 100%]. For **(B)** and **(C)**, data are given as means ± SD (n = 10). Means with different letters are significantly different (P < 0.05, Tukey’s test).

The accumulation of OsTCTP was obviously induced by HgCl_2_ stress (Figure 7A); therefore, *OsTCTP*-OX and *OsTCTP*-RNAi transgenic plants were evaluated for Hg tolerance. When WT (cv. ‘Nipponbare’), VC (vector control), *OsTCTP*-RNAi (#10 and #17) and *OsTCTP*-OX (#12 and #14) were grown in the absence of Hg, their root growth was similar (Figure 8A). However, in the presence of 0.2 μM Hg, the root growth was inhibited more in the *OsTCTP*-RNAi than in the WT, VC or *OsTCTP*-OX transgenic plants (Figure 8A). For the *OsTCTP*-RNAi transgenic plants, the root elongation was inhibited by approximately 61%, whereas the inhibition rates were only around 52% and 37% for the control plants (WT and VC) and the *OsTCTP*-OX transgenic plants, respectively (Figure 8B,C). Synchronously, 6-week-old rice seedlings grown hydroponically were also subjected to Hg stress. The *OsTCTP*-RNAi transgenic plants displayed a severe leaf curl phenotype after being treated with 50 μM HgCl_2_ for 3 days compared with the *OsTCTP*-OX transgenic plants and the control plants (WT and VC, Additional file 1: Figure S6). These results demonstrated that OsTCTP positively regulates Hg tolerance in rice.

### Hg and GSH contents in the transgenic and wild-type rice plants under Hg treatment

**Figure 9:**
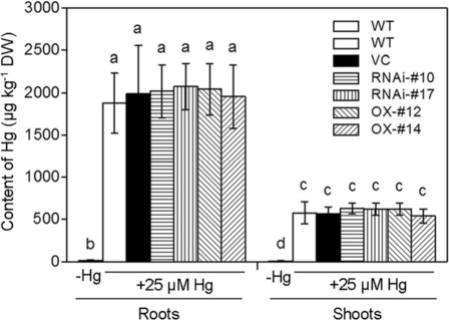
Mercury concentrations in roots or leaves of the wild type (WT), vector control (VC), *OsTCTP*-OX and *OsTCTP*-RNAi transgenic rice seedlings under 25 μM HgCl_2_ treatment for 3 days. Data are given as the mean ± SD of three biological replicates. Means with different letters are significantly different (P < 0.05, Tukey’s test).

**Figure 10:**
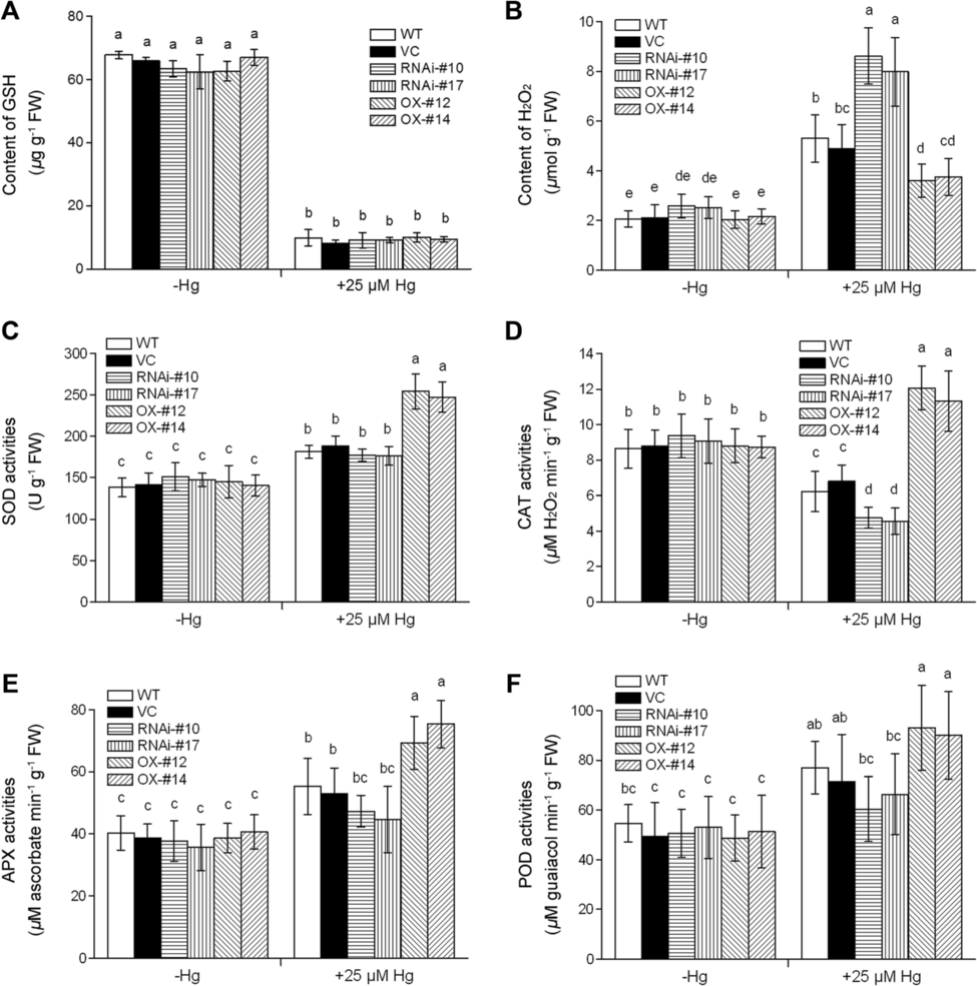
Analysis of GSH content, H_2_O_2_ level and antioxidant enzyme activities in roots of the wild type (WT), vector control (VC), *OsTCTP*-OX and *OsTCTP*-RNAi transgenic rice plants. **(A)** The total GSH content in roots of WT (cv. ‘Nipponbare’), VC (vector control), *OsTCTP*-RNAi (#10 and #17) and *OsTCTP*-OX (#12 and #14). **(B)** The H_2_O_2_ levels in roots of WT (cv. ‘Nipponbare’), VC (vector control), *OsTCTP*-RNAi (#10 and #17) and *OsTCTP*-OX (#12 and #14). **(C)** Superoxide dismutase (SOD) activity. **(D)** Catalase (CAT) activity. **(E)** Ascorbate peroxidase (APX) activity. **(F)** Peroxidase (POD) activity. Data are given as means ± SD of three biological replicates. Means with different letters are significantly different (P < 0.05, Tukey’s test).

To determine whether the overexpression of *OsTCTP* prevents Hg absorption or accumulation, we measured the Hg content in roots and shoots of the *OsTCTP*-RNAi and *OsTCTP*-OX transgenic rice plants and the control plants (WT and VC). After being treated with 25 μM HgCl_2_ for 3 days, Hg residue was significantly increased in both roots and shoots of the transgenic and control rice plants. However, the concentration of Hg in neither roots nor shoots of the transgenic lines was different from that in the control rice plants (Figure 9), indicating that the overexpression of *OsTCTP* did not prevent Hg absorption and transport. Furthermore, the glutathione (GSH)-dependent phytochelatin (PC) synthesis pathway is one of the most important mechanisms contributing to divalent heavy metal detoxification and tolerance [38,39], so we determined the GSH content in roots of the *OsTCTP*-RNAi and *OsTCTP*-OX transgenic rice plants and the control plants (WT and VC). The level of total GSH in roots was greatly reduced with 25 μM HgCl_2_ for 12 hours, and the decreased level of GSH caused by Hg stress in the *OsTCTP*-RNAi and *OsTCTP*-OX transgenic rice plants showed no difference from that in the control plants (WT and VC) (Figure 10A), which is consistent with previous report [40]. At the transcriptional level, we also tested the expression of relative genes, such as two glutathione synthetase genes (Os12g34380 and Os11g42350), two phytochelatin synthase genes (Os05g34290 and Os06g01260) and an ABCC-type transporter 1 gene (Os04g52900), but none of them had significantly altered transcripts between the transgenic rice plants (*OsTCTP*-RNAi and *OsTCTP*-OX lines) and the control plants (WT and VC) under normal and Hg treatment (Additional file 1: Figure S7). These results indicated that rice probably does not employ the PC synthesis pathway to mediate Hg tolerance and the OsTCTP does not affect Hg absorption and accumulation in rice.

### H_2_O_2_ level and changes of antioxidant enzyme activity in response to Hg stress

A growing number of studies have shown that mercuric ions are able to induce oxidative stress in plants [4,11,12,41]. This prompted us to investigate whether ROS accumulation differentially occurred in roots of the *OsTCTP*-RNAi and *OsTCTP*-OX transgenic rice plants under Hg stress by quantifying the accumulation of H_2_O_2_. These results indicated that, under Hg stress condition, *OsTCTP*-OX transgenic lines showed significantly less accumulation of H_2_O_2_ compared to the control plants (WT and VC) and the *OsTCTP*-RNAi transgenic lines (Figure 10B), suggesting that OsTCTP may be involved in ROS homeostasis in rice plants in response to Hg toxicity. To further elucidate the mechanism of ROS detoxifying machinery under Hg stress condition, the activities of antioxidant enzymes, such as superoxide dismutase (SOD), catalase (CAT), ascorbate peroxidase (APX), and peroxidase (POD) were also analyzed. The activities of SOD, CAT, APX, and POD increased rapidly in Hg stress-treated *OsTCTP*-OX transgenic lines compared with the control plants (WT and VC) and the *OsTCTP*-RNAi transgenic lines (Figure 10C–F), especially, in the case of CAT (Figure 10D). These results suggested that the antioxidant enzyme system is strongly promoted in the *OsTCTP*-OX transgenic rice plants under Hg treatment.

## Discussion

### TCTPs are conserved and play vital roles in eukaryotic organisms

Sequence comparison shows that OsTCTP, like other TCTPs, contains a series of conserved functional domains for interaction with other proteins, such as the antiapoptotic protein MCL/BCL [27,42], the cell cycle controlling polo kinase [27,43], and a Na^+^/K^+^ ATPase [27,44] (Figure 3A). A Ca^2+^ binding domain, a TCTP self-interaction domain and a protein transduction domain have also been identified among different TCTPs [45-47]. Furthermore, our structural model for the OsTCTP shows high similarity to the human TCTP protein (Figure 3A,B), also suggesting that TCTPs from different species are strictly conserved in both amino acids and protein structure. TCTPs are ubiquitously expressed in all eukaryotic organisms and are related to various stress responses. It has been established that TCTP levels are highly regulated in response to a wide range of extracellular and intracellular signals, and various stress conditions such as starvation, heat shock, heavy metals, calcium stress or pro-apoptotic signals [24]. In *Drosophila*, disruption of *dTCTP* expression in an organ-specific manner leads to size reduction of the targeted organ due to a reduction in cell numbers and defects in cell growth [48]. Interestingly, plant TCTP can fully rescue cell proliferation defects in *Drosophila* loss of function for dTCTP [25], indicating that TCTPs are functionally conserved between plant and animal species.

### OsTCTP is required for Hg tolerance in rice

*OsTCTP* is ubiquitously expressed in all tested plant tissues (Figure 5), whose expression is rapidly induced by Hg at both mRNA level (Additional file 1: Figure S8) and protein level (Figure 7A). Overexpression of *OsTCTP* resulted in increased tolerance to Hg, while knockdown of *OsTCTP* by RNA interference led to sensitivity to Hg (Figure 8). These results indicate that *OsTCTP* is required for Hg tolerance in rice. Knockdown of *OsTCTP* led to a huge increase in Hg-induced H_2_O_2_ in RNA interference lines under Hg stress condition, whereas overexpression of *OsTCTP* had opposite effect (Figure 10B). Overexpression of *OsTCTP* promoted the activities of SOD, CAT, APX, and POD (Figure 10C–F), and decreased the Hg-induced H_2_O_2_ in overexpression lines under Hg stress condition. These results suggested that OsTCTP functions to enhance tolerance to Hg stress through the regulation of ROS and their scavengers, although these regulating mechanisms for activities of antioxidant enzymes remain to be molecularly characterized.

### OsTCTP is not an N-glycoprotein

It has been shown that the protein accumulation and potential glycosylation of TCTP is regulated by glucose in pancreatic beta cells [49]. Therefore, we predicted the potential glycosylation sites in OsTCTP sequence on NetNGlyc website (http://www.cbs.dtu.dk/services/NetNGlyc/) and N48 (Asn 48) and N97 (Asn 97) were forecasted to be the potential N-glycosylation sites (Additional file 1: Figure S9A). Previous reports have demonstrated that the mobility shift will change and result in a small band when the polysaccharide chain is released from the glycoprotein [50,51]. To confirm the N-glycosylation sites of OsTCTP, we performed site-directed mutagenesis of the two potential N-glycosylation sites by substituting Asn with Gln (N48Q and N97Q; Additional file 1: Figure S9B). The mutated genes were inserted into the HA-tagged expression vectors and transiently expressed in *Nicotiana benthamiana* leaves as described by Sparkes *et al.* [52]. Western blot analysis showed that there was no difference in mobility shift of wild-type OsTCTP and its mutants (Additional file 1: Figure S9C). Altogether, these results suggested that N48 and N97 are not the potential N-glycosylation sites and OsTCTP is not an N-glycoprotein.

## Conclusions

In this study, we molecularly and functionally characterized a rice gene (Os11g43900, *OsTCTP*) and our data establish that OsTCTP plays a critical role in Hg tolerance by regulating ROS production and scavenging and overexpression of OsTCTP increases Hg resistance, whereas knockdown of OsTCTP has an opposite effect. In addition, further research is needed to understand how the OsTCTP regulates the activities of antioxidant enzymes in Hg stress signaling.

## Methods

### Plant materials and growth conditions

A wild-type rice (*Oryza sativa* L.) cultivar ‘Nipponbare’ belonging to sp. *japonica* was used in this study. The wild-type and transgenic plants were grown either in a greenhouse [average temperature of 28/24°C (day/night), relative humidity 60–80%, photosynthetically active radiation 200–400 μmol·m^−2^s^−1^ and photoperiod of 14 h day/10 h night] or in an experimental field at the Institute of Plant Sciences, Zhejiang University (Hangzhou, China).

### Plasmid construction and plant transformation

All constructs were made using standard procedures [53]. A universal expression vector pCUN1301 which carries a maize (*Zea mays*) ubiquitin 1 (Ubi1) promoter and a Nos terminator cassette was constructed as previously described by Chen *et al.* [54]. To make the overexpression constructs, two putative CDSs of *OsTCTP* were amplified by PCR using primer pairs (Additional file 2: Table S1) and subcloned into the plasmid pCUN1301 to result in the overexpression constructs (*OsTCTPa*-OX and *OsTCTPb*-OX). The *OsTCTPa*-OX and *OsTCTPb*-OX constructs were confirmed by sequencing. To make a dsRNAi construct, DNA fragments (508 bp, including 119 bp intact 5’UTR and 389 bp coding sequence) with different restriction enzyme sites at both ends was amplified by PCR using RNAi1 and RNAi2 primer pairs (Additional file 2: Table S1). The two PCR fragments were inserted at inverted repeats into the pKANNIBAL vector to generate a hairpin RNAi construct [55], which was then subcloned into plasmid pCUN1301 to result in the RNAi construct (*OsTCTP*-RNAi).

The *Agrobacterium*-mediated transformation was performed using vigorously growing calli derived from mature embryos of rice (cv. ‘Nipponbare’) following a standard procedure [32].

### PCR selection, southern blot analysis and GUS staining

The transgenic plants were regenerated from transformed calli by selecting for hygromycin resistance. The regenerated plants were confirmed by PCR analysis using the hygromycin phosphotransferase-specific primer (Additional file 2: Table S1). Plant genomic DNAs were prepared from leaves of rice leaves using cetyltrimethylammonium bromide [56] and were quantified using SMA4000 UV–vis Spectrophotometer (Merinton). Thirty micrograms of DNA were digested with *Eco*RI; electrophoresed on 0.8% agarose gel; blotted on a nylon membrane (Roche) with a high-salt buffer [57]. A hygromycin phosphotransferase probe was generated using the DIG-High Prime DNA Labeling and Detection Starter Kit I or II (Roche), and the hybridization signals were detected according to the standard manufacturer’s instructions. GUS staining was conducted essentially as previously described by Jefferson *et al.* [58].

### Protein purification and antibody production

A gene fragment between +1 and +360 (related to the ATG at +1 bp) was amplified from the *OsTCTP* coding sequence by PCR and then inserted into the *Nde*I and *Xho*I restriction sites of the pET-28a (+) to generate the expression vector (*OsTCTP*-28a). The plasmid encoding the His-fusion protein was transformed into *Escherichia coli* BL21 (DE3) strain (Novagen). When the culture of the transformed cells reached an A_600_ of 0.8–1.0, protein accumulation was induced by 1 mM isopropyl 1-thio-β-D-galactopyranoside (IPTG) at 20°C for 4–6 h. The His-taged protein was purified by using His•Bind purification kit (Novagen) according to the manufacturer’s protocol. The quality of the purified protein was assessed by SDS-PAGE and coomassie brilliant blue (CBB) staining. The buffer with the highly purified OsTCTP protein was exchanged to 1× PBS using a dialyzer. This antigen solution (5 mg mL^−1^ protein) was injected into rabbits using standard protocols for antibody production [53], and the resulting antiserum was used for western blot.

### Protein extraction and western blot analysis

Samples (approximately 0.1 g) were ground to powder with liquid nitrogen and homogenized in 400 μL extraction buffer [50 mM Tris–HCl (PH 7.5), 150 mM NaCl, 5 mM EDTA, 0.5% Sodium deoxycholate, 0.5% Triton X-100, 0.1% SDS, 0.2% Nonidet P-40, 1 mM PMSF, 5 mM DTT and 10% Glycerol]. The homogenates were shaken repeatedly on ice for 30 min and centrifuged at 16,000× *g* for 20 min at 4°C, and the resulting supernatants were used for western blot analysis. Twenty micrograms of supernatants were mixed with 5 μL of 4× SDS sample buffer [250 mM Tris–HCl (pH 6.8), 8% SDS, 40% glycerol, 0.4% Bromophenol blue and 6% β-mercaptoethanol], and then incubated at 85°C for 10 min. The samples were then analyzed by 4%–12% (w/v) SDS–PAGE. After electrophoresis, proteins were electroblotted to nitrocellulose membranes. Membranes were blocked in 5% nonfat dried milk in TBS-T [20 mM Tris–HCl (pH 7.4), 150 mM NaCl and 0.05% Tween 20] overnight at 4°C and then washed for 3× 10 min in TBS-T. The membranes were incubated with the rabbit OsTCTP polyclonal antibody (1:2500) or with the β-actin mouse monoclonal antibody (1:1000) diluted with 1% BSA in TBS-T for 1.5 h at room temperature. Next, the membranes were washed in TBS-T for 3× 10 min, and incubated for 1 h with goat anti-rabbit IgG or goat anti-mouse IgG (1:5000 dilution in TBS-T with 1% BSA) conjugated to horseradish peroxidase, followed by a washing for 3× 10 min in TBS-T. Finally, chemiluminescent signals were detected using Luminata Forte Western HRP substrate (Millipore). The quantitative assessment was processed by Quantity One® Software (http://www.bio-rad.com).

### RNA extraction and qRT-PCR analysis

Total RNA was extracted from materials using RNAprep Pure Plant Kit (Tiangen), and cDNA was reverse transcribed from 1 μg of total RNA using M-MLV reverse transcriptase (Takara). Relative quantification of gene expression by qRT-PCR was performed on a LightCycler® 480 II instrument (Roche). qRT-PCR was performed in an optical 384-well plate, including 10 μL 2× SYBR Green Master mix reagent (Takara), 1 μL 1:10-diluted template cDNA, and 0.2 μM of each gene-specific primers, in final volume of 20 μL, using the thermal cycles as follows: 95°C for 1 min; 40 or 45 cycles of 95°C for 10s; 55°C for 15s; and 72°C for 20s. Disassociation curve analysis was performed as follows: 95°C for 15s; 60°C for 15s; 94°C for 1 min. The relative expression levels were calculated by the ΔC_T_ method [59]. Rice *histoneH3* gene was used as endogenous control which was stable throughout the Al treatment [60]. The primers used for studying gene expression are listed in Additional file 2: Table S1. The reactions were performed in triplicate, and the results were averaged.

### Isolation and analysis of T-DNA insertion lines

The rice T-DNA insertion mutant *ostctp* was identified from the Rice T-DNA Insertion Sequence Database (http://cbi.khu.ac.kr) [36], Kyung Hee University, Korea. The homozygote and heterzygote screening was performed using PCR as described previously [37]. qRT-PCR and western blot were performed to determine knockdown or knockout of the *OsTCTP* transcripts and translation products, respectively.

### Transient expression of the fused sGFP protein

To make a GFP construct, the OsTCTP coding region was fused to sGFP and subcloned into the pCAMBIA1300 vector with a 35S promoter to generate the GFP construct (*OsTCTP*-GFP). The DNA constructs were introduced into onion (*Allium cepa*) epidermal cells as described previously [61].

### Chemical and abiotic treatments

Chemical treatments and mechanical wounding were conducted essentially as previously described by Xiong & Yang [62]. For chemical treatments, roots of 2-week-old seedlings were immersed in solutions containing abscisic acid (ABA, 0.1 mM), jasmonic acid (JA, 0.1 mM), salicylic acid (SA, 1 mM), 1-naphthylacetic acid (NAA, 0.1 mM), or hydrogen peroxide (H_2_O_2_, 100 mM) for 0 to 48 hours under the greenhouse conditions. Mechanical wounding was achieved by crushing rice leaves with a hemostat. Abiotic treatments were conducted mainly according to Saijo *et al.* [63] and Jang *et al.* [64]. For the salt stress treatment, roots of 2-week-old seedlings were immersed in 200 mM NaCl solution for 0 to 48 hours under the greenhouse conditions. For heavy metal stress, roots of 2-week-old seedlings were immersed in solutions containing HgCl_2_ (25 μM) or CuSO_4_ (50 μM) for 0 to 48 hours under the greenhouse conditions, respectively. Drought stress was induced by 15% polyethylene glycol (PEG) 6000 (150 g L^−1^) solution for 0 to 48 hours under the greenhouse conditions.

### Evaluation of the Hg tolerance and determination of Hg content

For evaluation of the Hg tolerance, seeds of WT (cv. ‘Nipponbare’) and T2 transgenic seeds of VC (vector control), *OsTCTP*-RNAi (#10 and #17) and *OsTCTP*-OX (#12 and #14) were germinated in water and solution containing 25 mg L^−1^ hygromycin B (Roche), respectively, at 28°C for 3 days, and then transferred to a plastic net floating on 0.5 mM CaCl_2_ solution (pH 5.5) containing 0 or 0.2 μM HgCl_2_. After 7 days, the root length was measured using a ruler. The inhibition rate was defined as [1 – (the ratio of the root elongated of the plants receiving Hg treatment to that of the no-Hg control) × 100%]. 2-week-old rice seedlings of WT (cv. ‘Nipponbare’), VC (vector control), *OsTCTP*-RNAi (#10 and #17) and *OsTCTP*-OX (#12 and #14) treated with or without 25 μM HgCl_2_ for 3 days were used for the determination of the Hg concentration. The sample preparation and Hg determination was performed as described previously [65,66]. Briefly, the dried sample (0.1 g) was submerged in 5 mL of the acidic oxidative mixture (HNO_3_:H_2_O_2_:H_2_O; 0.6: 0.4: 1; v/v), and autoclave digested (120°C, 1.5 atmospheres, 30 min). Once cooled to room temperature, the digests were filtered through a polyvinylidenefluoride (PVDF) filter and diluted in water to 50 mL. Hg was analysed using a flux injection absorption spectrometer, equipped with the cold-vapour generator FIAS (Perkin Elmer, USA).

### Measurement of the GSH, H_2_O_2_ and antioxidant enzyme assays

For VC (vector control), *OsTCTP*-RNAi (#10 and #17) and *OsTCTP*-OX (#12 and #14) transgenic plants, seeds of T2 generation selected with 25 mg L^−1^ hygromycin B (Roche) were employed for the experiments. 2-week-old rice seedlings of WT (cv. ‘Nipponbare’), VC (vector control), *OsTCTP*-RNAi (#10 and #17) and *OsTCTP*-OX (#12 and #14) treated with or without 25 μM HgCl_2_ for 12 hours were used for measurement of the GSH and H_2_O_2_ contents as described previously [67-69]. For the determination of GSH, roots of rice plants (0.1 g fresh weight) were ground with liquid nitrogen and homogenized in 1 mL cold 5% trichloroacetic acid. After centrifugation at 4°C and 10,000× *g* for 10 min, the supernatant was used to measure the content of GSH according to the instruction of the total GSH assay kit (Beyotime, China). For H_2_O_2_ measurement, roots of rice plants (0.2 g fresh weight) were homogenized in 1 mL cold acetone in a mortar with silica sand. The extract and washings were centrifuged at 3,000× *g* for 15 min and the coloring materials were adsorbed by activated carbon. Then 200 μL supernatant were added to 1 mL of reaction buffer [0.25 mM FeSO_4_, 0.25 mM (NH4)_2_SO_4_, 25 mM H_2_SO_4_, 1.25 mM xylenol orange, and 1 mM sorbitol] at room temperature for 1 h. The level of H_2_O_2_ was estimated spectrophotometrically at 560 nm using a spectrophotometer. The results were calculated on the basis of a standard curve using standard hydrogen peroxide solutions.

For antioxidant enzyme assays, 2-week-old rice seedlings of WT (cv. ‘Nipponbare’), VC (vector control), *OsTCTP*-RNAi (#10 and #17) and *OsTCTP*-OX (#12 and #14) treated with or without 25 μM HgCl_2_ for 12 h were used for antioxidant enzyme assays. Roots of rice plants (0.2 g fresh weight) were homogenized and extracted in 1 mL of ice cold 50 mM sodium phosphate buffer (pH 7.8) containing 1 mM EDTA at 4°C. After centrifugation at 13,000 rpm for 10 min, the supernatant was used as a crude enzyme extract. The activity of superoxide dismutase (SOD), catalase (CAT), peroxidase (POD) and ascorbate peroxidase (APX) was determined as described previously [70].

### Site-directed mutagenesis and N-glycosylation analysis

To make mutant OsTCTP proteins, N48Q and N97Q changes were made by site-directed mutagenesis, using an in vitro synthesis method (Transgen). Each wild-type and mutated *OsTCTP* was subcloned into the transient expression vector pCHA at *Kpn*I and *Bam*HI sites, and the constructs were transformed into *Nicotiana benthamiana* via *Agrobacterium* EHA105-mediated transformation [48]. Protein extraction and western blot analysis were performed following a standard procedure as described above.

### Phylogenetic analysis and homology modelling

We constructed neighbor-joining (NJ) trees using the MEGA software (version 5.2) (http://www.megasoftware.net) [71] with the following parameters: Poisson correction, pairwise deletion, and bootstrap (1000 replicates; random seed). This was performed essentially as described by Zhang *et al.* [72].

The structure of the OsTCTP protein (Os11g43900) was modeled using the known structure of the human TCTP (PDB ID 2HR9, http://www.rcsb.org) as a template on the Swiss-Model workspace (http://swissmodel.expasy.org) [73].

### Statistical analysis

Data shown are mean ± standard deviation (SD) for at least three independent experiments. Mean differences were compared using the statistical software data processing system (DPS v7.05) [74], followed by the Tukey’s test and the difference at P < 0.05 and 0.01 is considered as significant and highly significant, respectively.

## Availability of supporting data

The data set supporting the results of this article is available in the National Center for Biotechnology Information repository, [GenBank: KR080533; http://www.ncbi.nlm.nih.gov/genbank/].

## Additional files

Additional file 1: Figures S1-S9.
**Figure S1.** Comparison of *OsTCTPa* and *OsTCTPb*. **Figure S2.** Western blot and southern blot analyses of *OsTCTPa* transgenic rice plants. **Figure S3.** Southern blot analysis of *OsTCTPb* transgenic rice plants. **Figure S4.** Protein accumulation profiles of OsTCTP under NaCl, MW, PEG, SA, JA or NAA treatments. **Figure S5.** Western blot and southern blot analyses of *OsTCTP*-RNAi transgenic rice plants. **Figure S6.** Phenotypic analysis of the wild type (WT), vector control (VC), *OsTCTP*-OX and *OsTCTP*-RNAi transgenic rice plants. Hg tolerance of WT (cv. Nipponbare), VC (vector control), *OsTCTP*-RNAi (#10 and #17) and *OsTCTP*-OX (#12 and #14). **Figure S7.** Expression patterns of some Hg stress-related genes in the wild type (WT), vector control (VC), *OsTCTP*-OX and *OsTCTP*-RNAi transgenic rice plants treated without or with 25 μM HgCl_2_ for 12 hours. **Figure S8.** qRT-PCR analysis of *OsTCTP* mRNA level in roots and shoots of the wild-type (WT) plants treated without or with 25 μM HgCl_2_ for 0 to 24 hours. **Figure S9.** Analysis of potential N-glycosylation sites in OsTCTP sequence. Additional file 2: Table S1. Oligonucleotide primers used in this study.

## Abbreviations

Hg: Mercury
TCTP: Translationally controlled tumor protein
ROS: Reactive oxygen species
TOR: Target of rapamycin
GFP: Green fluorescent protein
OX: Overexpression
RNAi: RNA interference
WT: Wild type
VC: Vector control
PEG: Polyethylene glycol
ABA: Abscisic acid
NAA: 1-Naphthaleneacetic acid
SA: Salicylic acid
JA: Jasmonic acid
GSH: Glutathione
PC: Phytochelatin
SOD: Superoxide dismutase
CAT: Catalase
APX: Ascorbate peroxidase
POD: Peroxidase
IPTG: 1-thio-β-D-galactopyranoside
CBB: Coomassie brilliant blue
SD: Standard deviation
