# Supplementary material for: OsTCTP, encoding a translationally controlled tumor protein, plays an important role in mercury tolerance in rice
Source: BMC Plant Biol. 2015 May 20;15:123. doi: 10.1186/s12870-015-0500-y (PMC4438481; doi:10.1186/s12870-015-0500-y)
Supplement: Additional file 1: Figures S1-S9. — Figure S1. Comparison of OsTCTPa and OsTCTPb. Figure S2. Western blot and southern blot analyses of OsTCTPa transgenic rice plants. Figure S3. Southern blot analysis of OsTCTPb transgenic rice plants. Figure S4. Protein accumulation profiles of OsTCTP under NaCl, MW, PEG, SA, JA or NAA treatments. Figure S5. Western blot and southern blot analyses of OsTCTP-RNAi transgenic rice plants. Figure S6. Phenotypic analysis of the wild type (WT), vector control (VC), OsTCTP-OX and OsTCTP-RNAi transgenic rice plants. Hg tolerance of WT (cv. Nipponbare), VC (vector control), OsTCTP-RNAi (#10 and #17) and OsTCTP-OX (#12 and #14). Figure S7. Expression patterns of some Hg stress-related genes in the wild type (WT), vector control (VC), OsTCTP-OX and OsTCTP-RNAi transgenic rice plants treated without or with 25 μM HgCl2 for 12 hours. Figure S8. qRT-PCR analysis of OsTCTP mRNA level in roots and shoots of the wild-type (WT) plants treated without or with 25 μM HgCl2 for 0 to 24 hours. Figure S9. Analysis of potential N-glycosylation sites in OsTCTP sequence. [file 12870_2015_500_MOESM1_ESM.doc]

# *OsTCTP*, encoding a translationally controlled tumor protein, plays an important role in mercury tolerance in rice

### Zhan Qi Wang^1^, Ge Zi Li^1^, Qiao Qiao Gong^1^, Gui Xin Li^2^, Shao Jian Zheng^1,§^

^1^State Key Laboratory of Plant Physiology and Biochemistry, College of Life Sciences, Zhejiang University, Hangzhou 310058, China

^2^College of Agronomy and Biotechnology, Zhejiang University, Hangzhou 310058, China

^§^Corresponding author**:** Shao Jian Zheng Ph.D.

### Additional file 1

**Figure S1.** Comparison of *OsTCTPa* and *OsTCTPb*. (A) Alignment of mRNAs of *OsTCTPa* and *OsTCTPb*. (B) Alignment of deduced amino acid sequences of *OsTCTPa* and *OsTCTPb*.

**Figure S2.** Western blot and southern blot analyses of *OsTCTPa* transgenic rice plants. (A) Western blot analysis of OsTCTP protein from transgenic plants using an antibody against rice OsTCTP. Equal amounts (20 μg) were loaded to each lane and were confirmed by coomassie brilliant blue (CBB) staining of Rubisco (bottom). The experiments were repeated for twice with similar results. (B) Southern blot analysis of the *HptII* gene. Total DNAs from *OsTCTPa* transgenic plants (30 μg for each lane) were digested with *Eco*RI and hybridized with the *HptII* gene-specific probe.

**Figure S3.** Southern blot analysis of *OsTCTPb* transgenic rice plants. Total DNAs from *OsTCTPb* transgenic plants (30 μg for each lane) were digested with *Eco*RI and hybridized with the *HptII* gene-specific probe.

**Figure S4.** Protein accumulation profiles of OsTCTP under NaCl, MW, PEG, SA, JA or NAA treatments. Western blot analysis of OsTCTP protein accumulation under different stress conditions. Hydroponically grown 2-week-old rice plants were transferred to solutions containing 200 mM NaCl (Na, A), mechanical wounding (MW, B), 15% PEG (C), 1 mM SA (D), 100 μM JA (E) or 100 μM NAA (F) for the time periods indicated. Equal amounts (20 μg) were loaded to each lane and were confirmed by coomassie brilliant blue (CBB) staining of Rubisco (bottom). The experiments were repeated for three times with similar results.

**Figure S5.** Western blot and southern blot analyses of *OsTCTP*-RNAi transgenic rice plants. (A) Western blot analysis of OsTCTP protein from transgenic plants using an antibody against rice OsTCTP. Equal amounts (20 μg) were loaded to each lane and were confirmed by coomassie brilliant blue (CBB) staining of Rubisco (bottom). The experiments were repeated for twice with similar results. (B) Southern blot analysis of the *HptII* gene. Total DNAs from *OsTCTP*-RNAi transgenic plants (30 μg for each lane) was digested with *Eco*RI and hybridized with the *HptII* gene-specific probe.

**Figure S6.** Phenotypic analysis of the wild type (WT), vector control (VC), *OsTCTP*-OX and *OsTCTP*-RNAi transgenic rice plants. Hg tolerance of WT (cv. Nipponbare), VC (vector control), *OsTCTP*-RNAi (#10 and #17) and *OsTCTP*-OX (#12 and #14). 6-week-old seedlings were exposed to nutrient solutions (pH 5.5) containing 0 (A) or 50 μM HgCl_2_ (B) for 3 days.

**Figure S7.** Expression patterns of some Hg stress-related genes in the wild type (WT), vector control (VC), *OsTCTP*-OX and *OsTCTP*-RNAi transgenic rice plants treated without or with 25 μM HgCl_2_ for 12 hours. qRT-PCR analysis of two glutathione synthetase genes, Os12g34380 (A) and Os11g42350 (B); two phytochelatin synthase genes, Os05g34290 (C) and Os06g01260 (D); an ABCC-type transporter 1 gene, Os04g52900 (E). *HistoneH3* was used as an internal standard and data are given as means ± SD of three biological replicates. Statistically significant differences in gene expression are indicated with different letters (P < 0.05, Tukey’s test).

**Figure S8.** qRT-PCR analysis of *OsTCTP* mRNA level in roots and shoots of the wild-type (WT) plants treated without or with 25 μM HgCl_2_ for 0 to 24 hours. *HistoneH3* was used as an internal standard and data are given as means ± SD of three biological replicates. Statistically significant differences in gene expression are indicated with asterisks: (*) p<0.05 or (**) p<0.01; Tukey’s test.

**Figure S9.** Analysis of potential N-glycosylation sites in OsTCTP sequence. (A) The prediction of potential N-glycosylation sites in OsTCTP sequence. (B) OsTCTP and its mutants. (C) Western blot analysis of WT and mutant OsTCTP proteins transiently expressed in tobacco leaves. The experiments were repeated for three times with similar results.

Figure S1


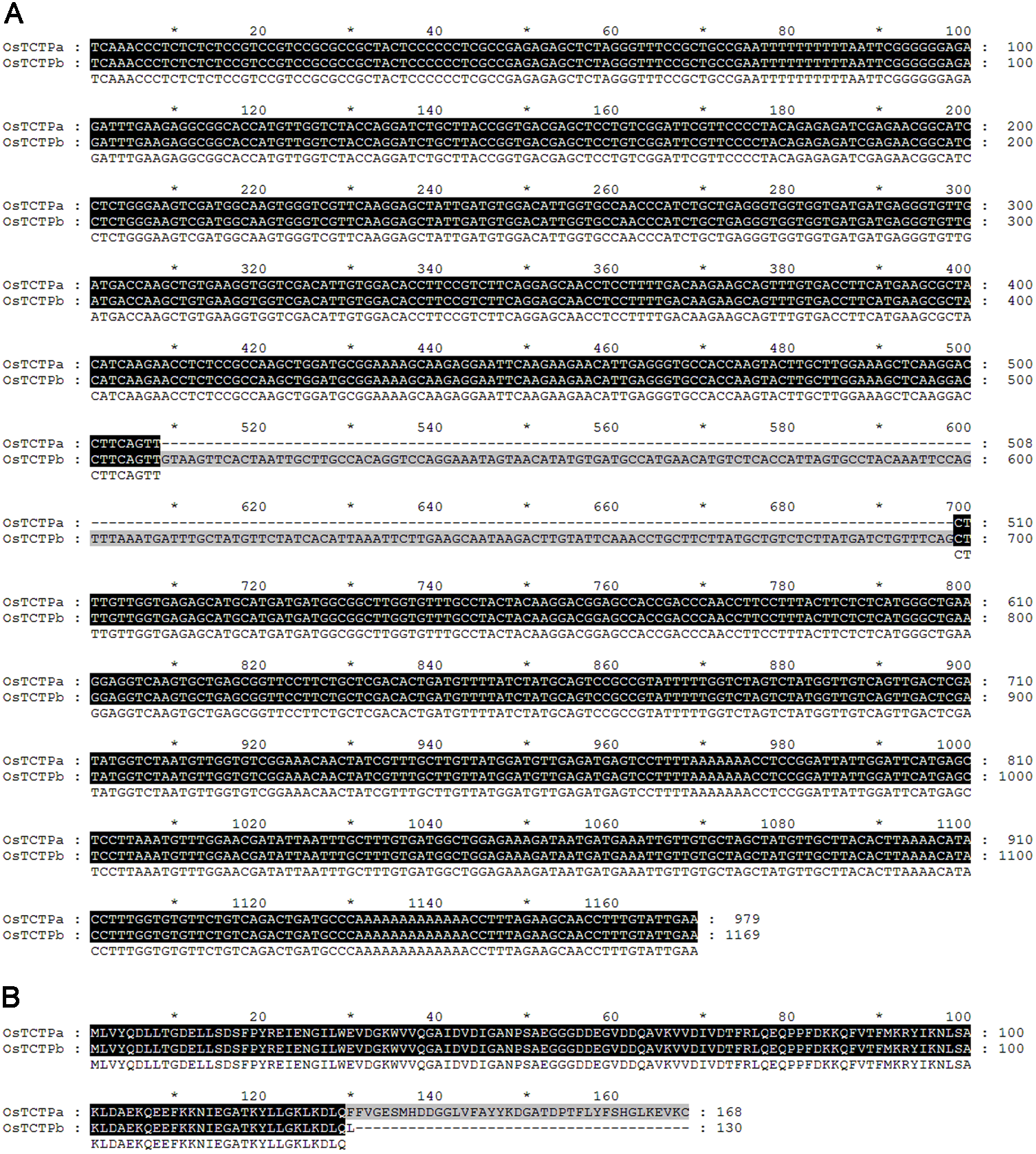


**Figure S1.** Comparison of *OsTCTPa* and *OsTCTPb*. (A) Alignment of mRNAs of *OsTCTPa* and *OsTCTPb*. (B) Alignment of deduced amino acid sequences of *OsTCTPa* and *OsTCTPb*.

Figure S2


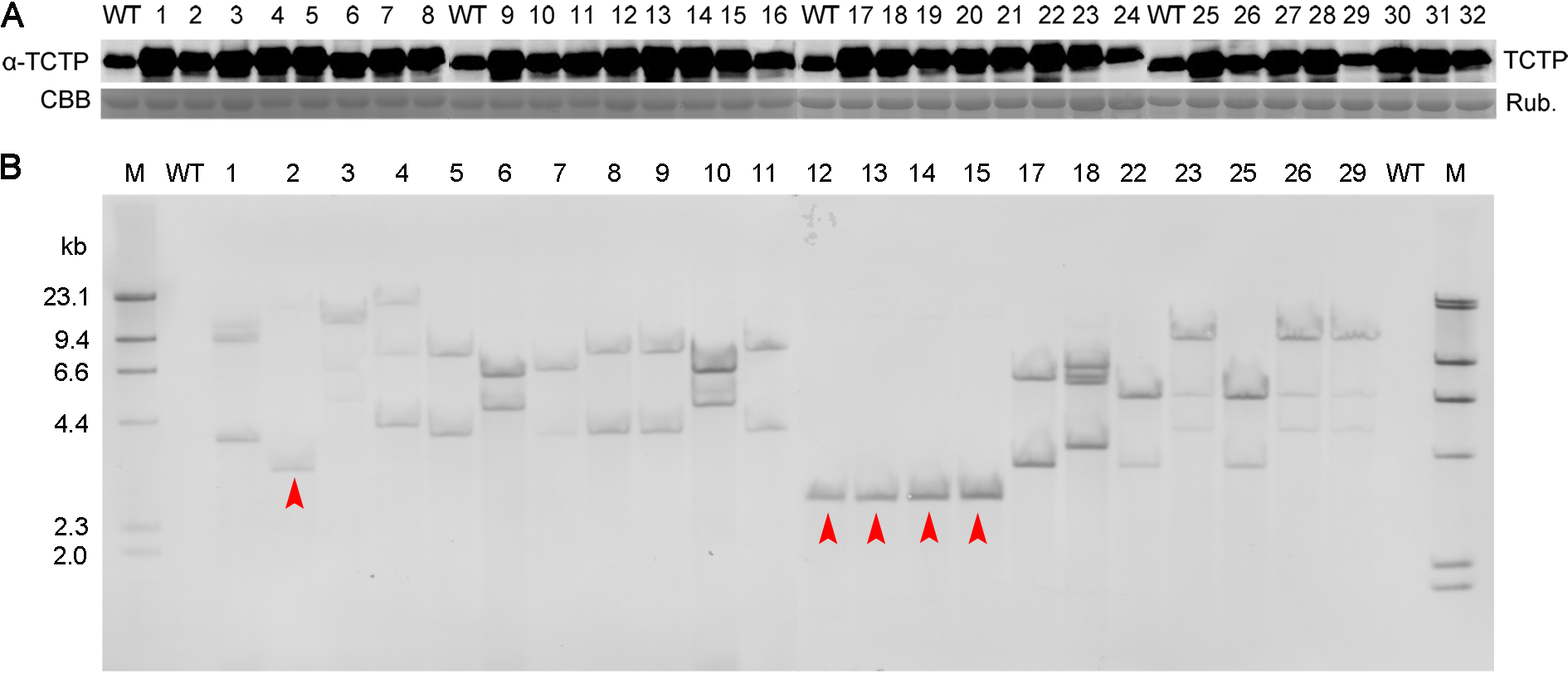


**Figure S2.** Western blot and southern blot analyses of *OsTCTPa* transgenic rice plants. (A) Western blot analysis of OsTCTP protein from transgenic plants using an antibody against rice OsTCTP. Equal amounts (20 μg) were loaded to each lane and were confirmed by coomassie brilliant blue (CBB) staining of Rubisco (bottom). The experiments were repeated for twice with similar results. (B) Southern blot analysis of the *HptII* gene. Total DNAs from *OsTCTPa* transgenic plants (30 μg for each lane) were digested with *Eco*RI and hybridized with the *HptII* gene-specific probe.

Figure S3


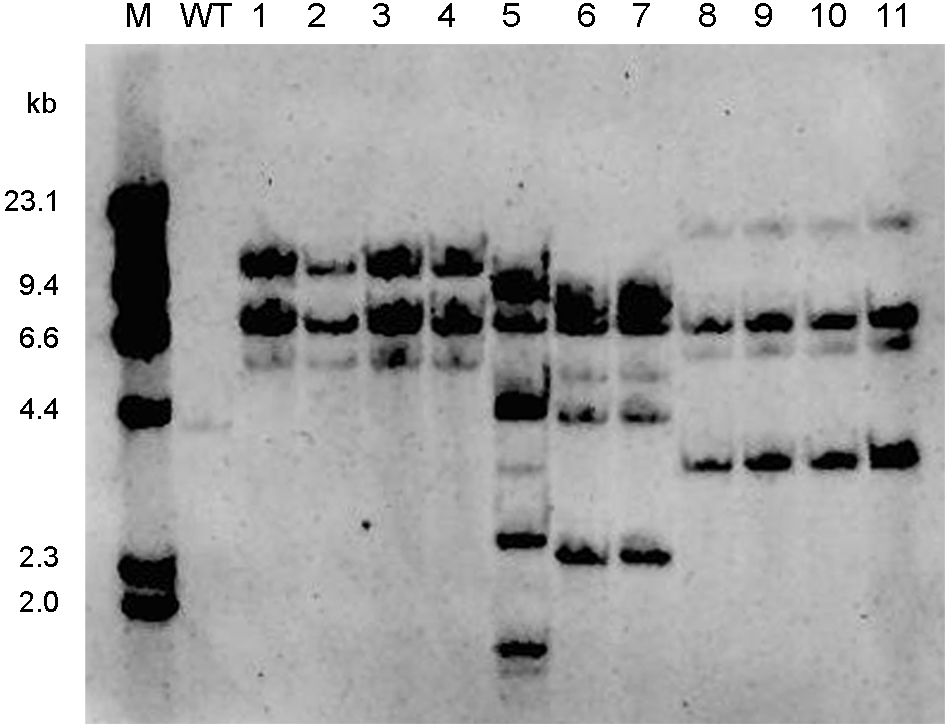


**Figure S3.** Southern blot analysis of *OsTCTPb* transgenic rice plants. Total DNAs from *OsTCTPb* transgenic plants (30 μg for each lane) were digested with *Eco*RI and hybridized with the *HptII* gene-specific probe.

Figure S4


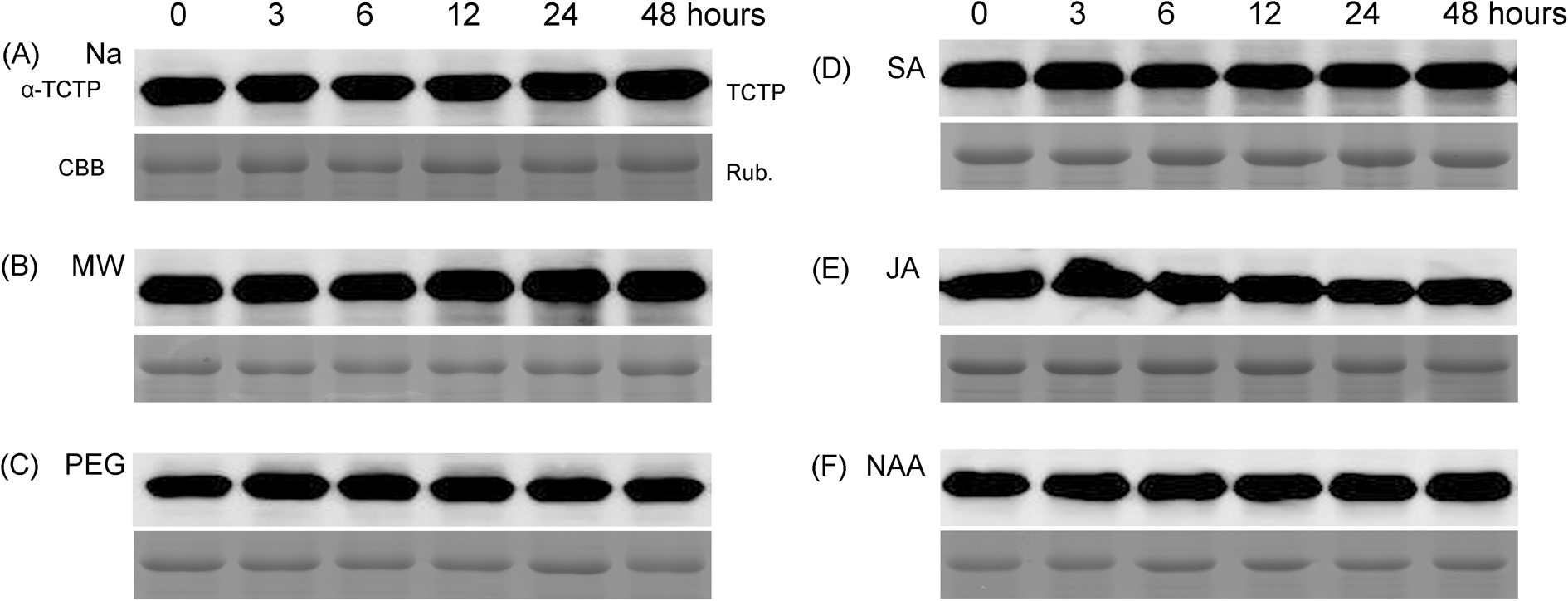


**Figure S4.** Protein accumulation profiles of OsTCTP under NaCl, MW, PEG, SA, JA or NAA treatments. Western blot analysis of OsTCTP protein accumulation under different stress conditions. Hydroponically grown 2-week-old rice plants were transferred to solutions containing 200 mM NaCl (Na, A), mechanical wounding (MW, B), 15% PEG (C), 1 mM SA (D), 100 μM JA (E) or 100 μM NAA (F) for the time periods indicated. Equal amounts (20 μg) were loaded to each lane and were confirmed by coomassie brilliant blue (CBB) staining of Rubisco (bottom). The experiments were repeated for three times with similar results.

Figure S5


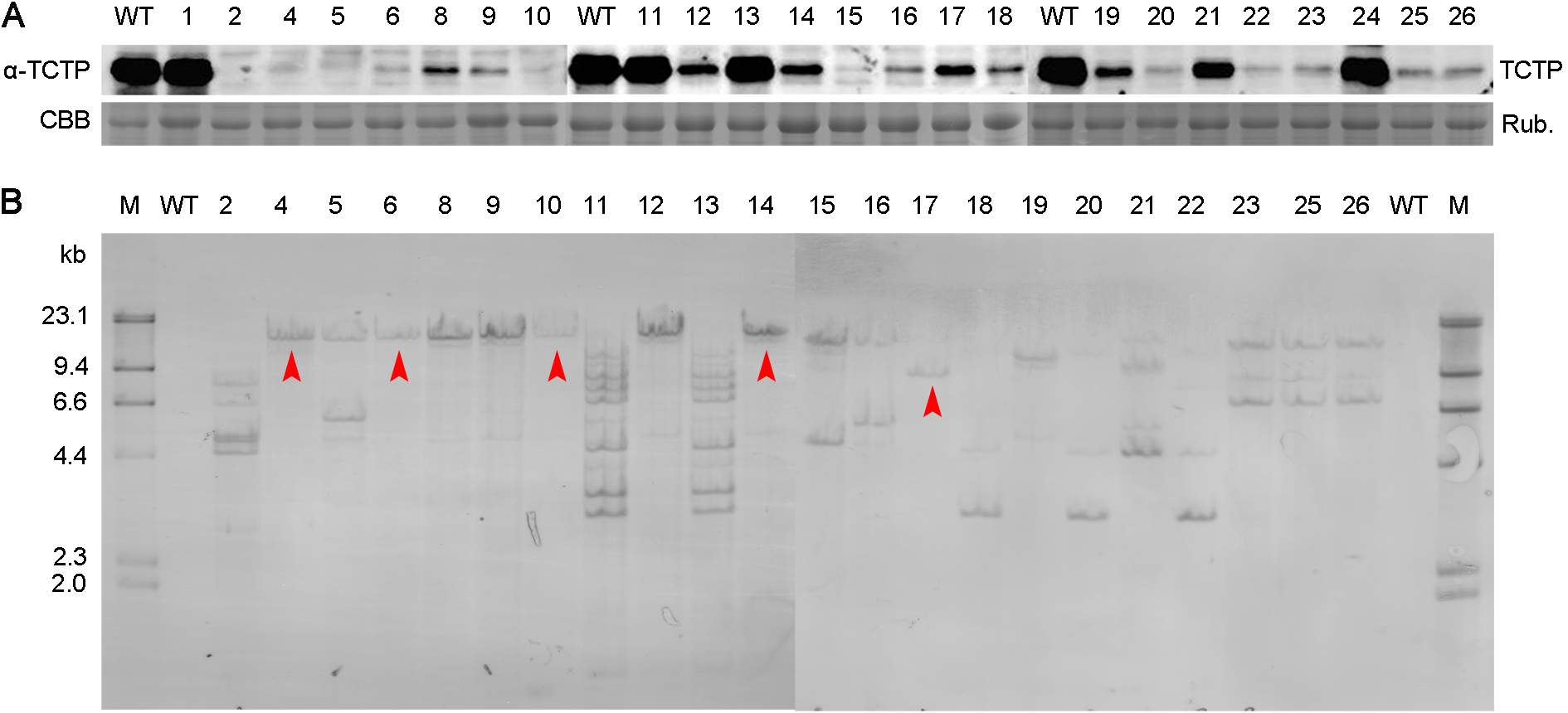


**Figure S5.** Western blot and southern blot analyses of *OsTCTP*-RNAi transgenic rice plants. (A) Western blot analysis of OsTCTP protein from transgenic plants using an antibody against rice OsTCTP. Equal amounts (20 μg) were loaded to each lane and were confirmed by coomassie brilliant blue (CBB) staining of Rubisco (bottom). The experiments were repeated for twice with similar results. (B) Southern blot analysis of the *HptII* gene. Total DNAs from *OsTCTP*-RNAi transgenic plants (30 μg for each lane) was digested with *Eco*RI and hybridized with the *HptII* gene-specific probe.

Figure S6


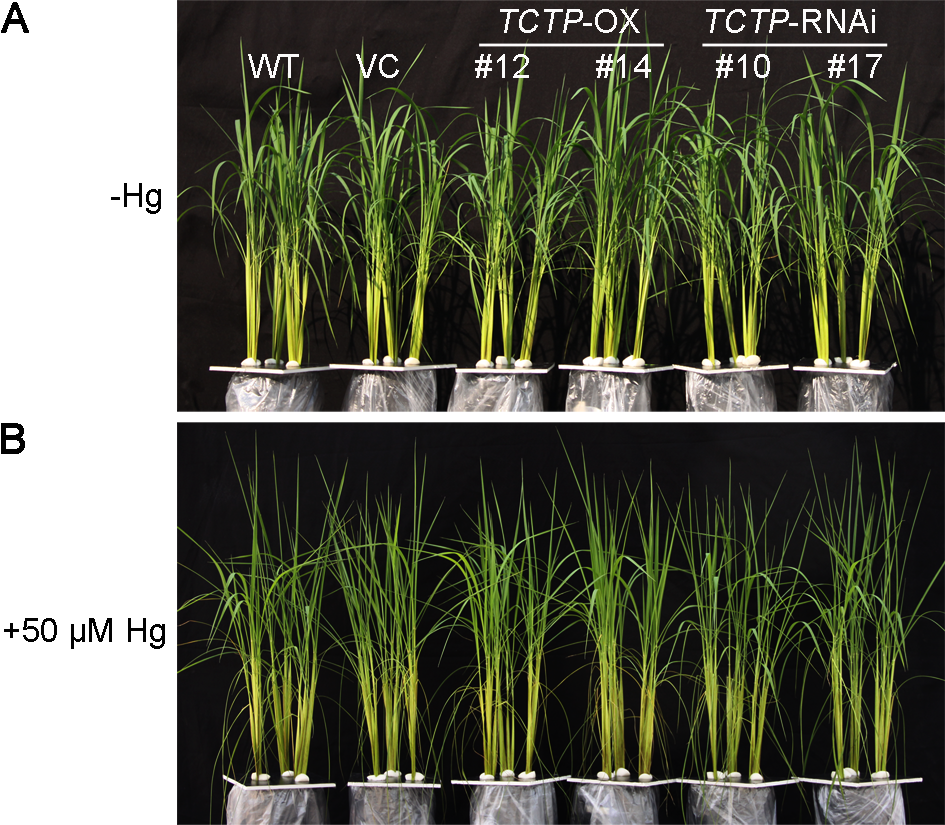


**Figure S6.** Phenotypic analysis of the wild type (WT), vector control (VC), *OsTCTP*-OX and *OsTCTP*-RNAi transgenic rice plants. Hg tolerance of WT (cv. Nipponbare), VC (vector control), *OsTCTP*-RNAi (#10 and #17) and *OsTCTP*-OX (#12 and #14). 6-week-old seedlings were exposed to nutrient solutions (pH 5.5) containing 0 (A) or 50 μM HgCl_2_ (B) for 3 days.

Figure S7


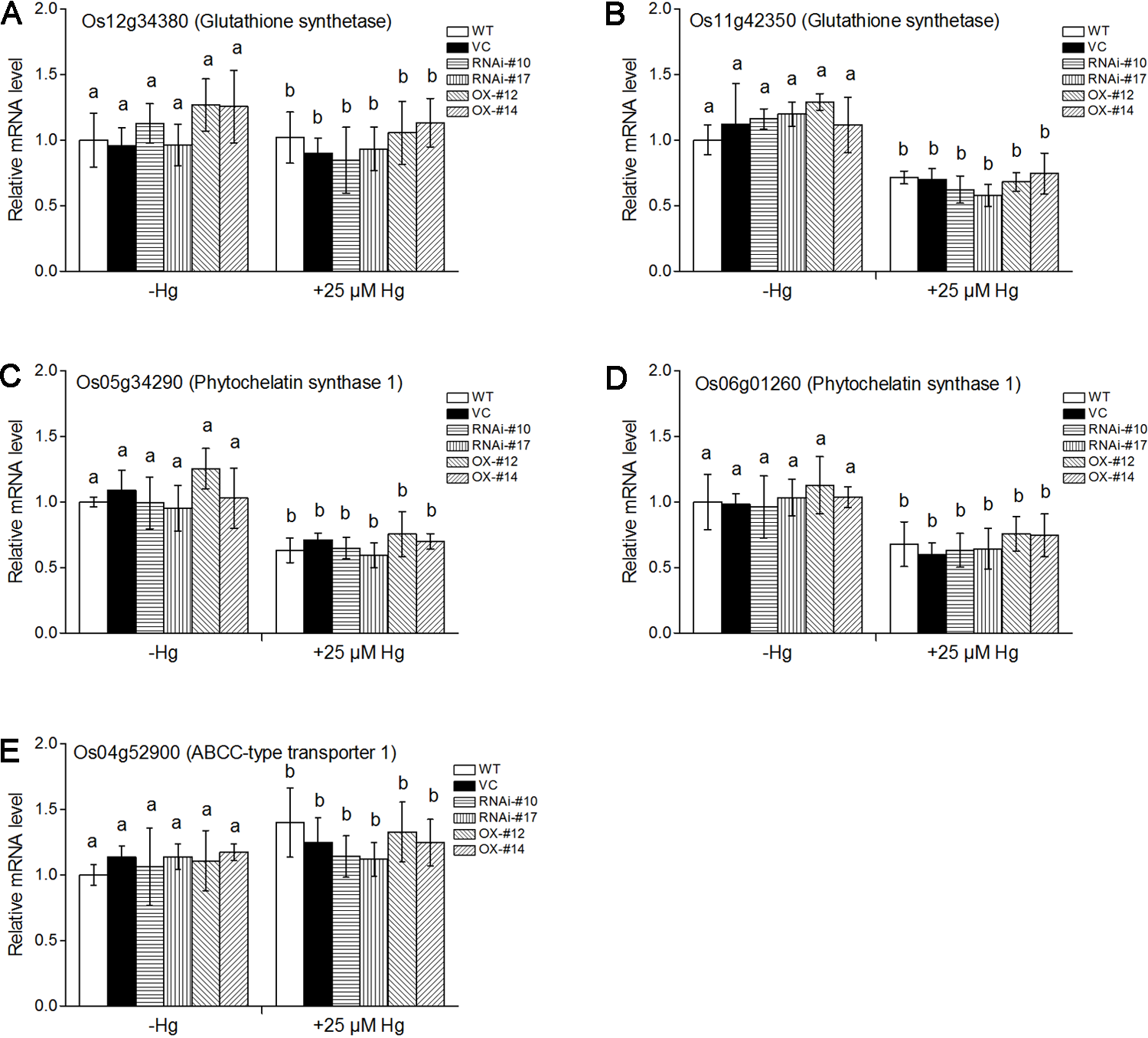


**Figure S7.** Expression patterns of some Hg stress-related genes in the wild type (WT), vector control (VC), *OsTCTP*-OX and *OsTCTP*-RNAi transgenic rice plants treated without or with 25 μM HgCl_2_ for 12 hours. qRT-PCR analysis of two glutathione synthetase genes, Os12g34380 (A) and Os11g42350 (B); two phytochelatin synthase genes, Os05g34290 (C) and Os06g01260 (D); an ABCC-type transporter 1 gene, Os04g52900 (E). *HistoneH3* was used as an internal standard and data are given as means ± SD of three biological replicates. Statistically significant differences in gene expression are indicated with different letters (P < 0.05, Tukey’s test).

Figure S8


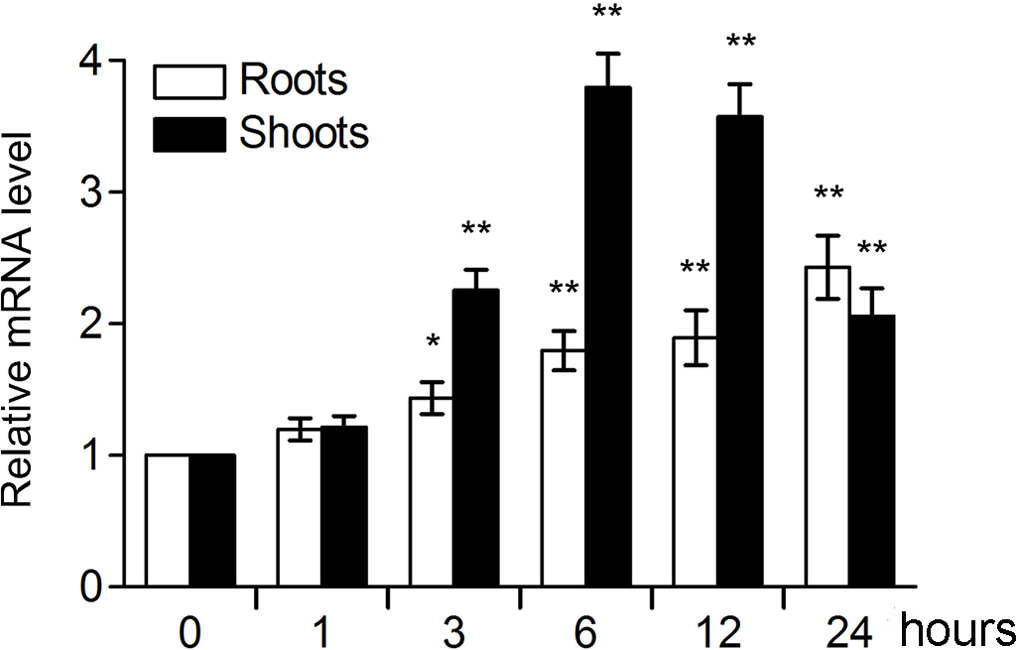


**Figure S8.** qRT-PCR analysis of *OsTCTP* mRNA level in roots and shoots of the wild-type (WT) plants treated without or with 25 μM HgCl_2_ for 0 to 24 hours. *HistoneH3* was used as an internal standard and data are given as means ± SD of three biological replicates. Statistically significant differences in gene expression are indicated with asterisks: (*) p<0.05 or (**) p<0.01; Tukey’s test.

Figure S9


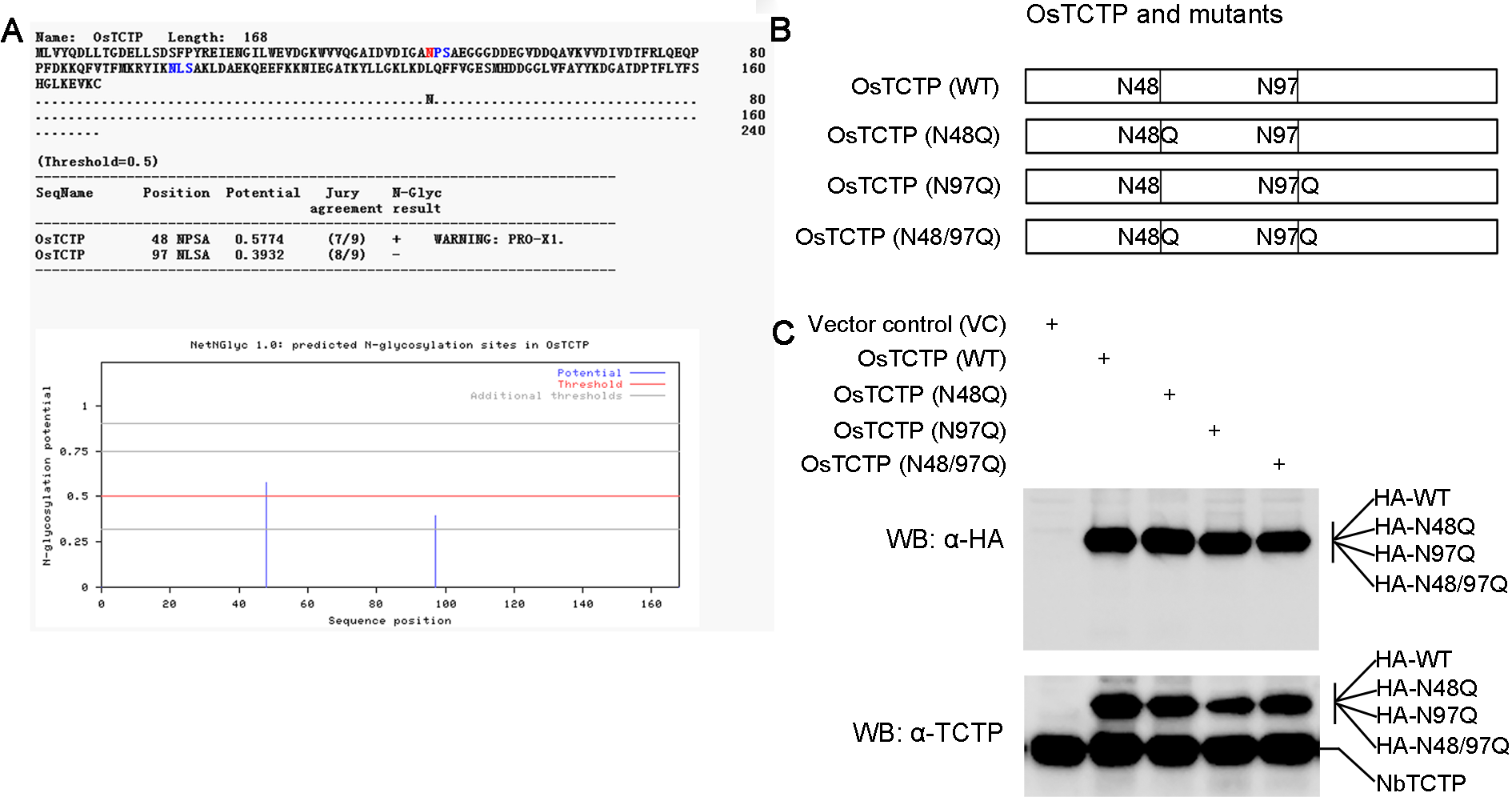


**Figure S9.** Analysis of potential N-glycosylation sites in OsTCTP sequence. (A) The prediction of potential N-glycosylation sites in OsTCTP sequence. (B) OsTCTP and its mutants. (C) Western blot analysis of WT and mutant OsTCTP proteins transiently expressed in tobacco leaves. The experiments were repeated for three times with similar results.
