# Supplementary material for: OsTCTP, encoding a translationally controlled tumor protein, plays an important role in mercury tolerance in rice
Source: BMC Plant Biol. 2015 May 20;15:123. doi: 10.1186/s12870-015-0500-y (PMC4438481; doi:10.1186/s12870-015-0500-y)
Supplement: Additional file 2: Table S1. — Oligonucleotide primers used in this study. [file 12870_2015_500_MOESM2_ESM.doc]

# *OsTCTP*, encoding a translationally controlled tumor protein, plays an important role in mercury tolerance in rice

### Zhan Qi Wang^1^, Ge Zi Li^1^, Qiao Qiao Gong^1^, Gui Xin Li^2^, Shao Jian Zheng^1,§^

^1^State Key Laboratory of Plant Physiology and Biochemistry, College of Life Sciences, Zhejiang University, Hangzhou 310058, China

^2^College of Agronomy and Biotechnology, Zhejiang University, Hangzhou 310058, China

^§^Corresponding author**:** Shao Jian Zheng Ph.D.

### Additional file 2

**Table S1.** Oligonucleotide primers used in this study.

**Table S1.** Oligonucleotide primers used in this study.

| Name | Sequence | Purpose |
| --- | --- | --- |
| TCTP-F | 5’-TGGATCCATGTTGGTCTACCAGGATCT | OsTCTPa/b-OX |
| TCTPa-R | 5’-TGGATCCTCAGCACTTGACCTCCTTC | OsTCTPa-OX |
| TCTPb-R | 5’-TGGATCCTTACAACTGAAGGTCCTTGA | OsTCTPb-OX |
| RNAi1-F | 5’-ACTCGAGGGATCCTCAAACCCTCTCTCTCCGTC | OsTCTP-RNAi |
| RNAi1-R | 5’-GCCGGTACCAACTGAAGGTCCTTGAGCTTTCC | OsTCTP-RNAi |
| RNAi2-F | 5’-ATCTAGAGGATCCTCAAACCCTCTCTCTCCGTC | OsTCTP-RNAi |
| RNAi2-R | 5’-GCCATCGATAACTGAAGGTCCTTGAGCTTTCC | OsTCTP-RNAi |
| TCTP-AbF | 5’-GTCCATATGTTGGTCTACCAGGATCT | OsTCTP-28a |
| TCTP-AbR | 5’-GCTCGAGTCAGCACTTGACCTCCTTC | OsTCTP-28a |
| TCTP-SlF | 5’-CAGGTACCATGTTGGTCTACCAGGATCT | OsTCTP-GFP |
| TCTP-SlR | 5’-AATCTAGAGCACTTGACCTCCTTCAGCC | OsTCTP-GFP |
| TCTP-GRF | 5’-TGAGGGTGGTGGTGATGATGAG | Genomic/RT-PCR |
| TCTP-GRR | 5’-CGCTCAGCACTTGACCTCCTTC | Genomic/RT-PCR |
| TCTPa/b-F | 5’-GTTGGTGAGAGCATGCATGATG | Probe |
| TCTPa/b-R | 5’-CAATACAAAGGTTGCTTCTAAAGGT | Probe |
| HptII-F | 5’-GAAAAAGCCTGAACTCACCGC | Probe |
| HptII-F | 5’-TGCTCCATACAAGCCAACCAC | Probe |
| FP | 5’-TTTTGCCCGATTCTTTTGTC | Genotyping |
| RP | 5’-CCTCTTCAAATCTCTCCCCC | Genotyping |
| RBP | 5’-AACGCTGATCAATTCCACAG | Genotyping |
| qH3-F | 5’-GGTCAACTTGTTGATTCCCCTCT | qRT-PCR |
| qH3-R | 5’-AACCGCAAAATCCAAAGAACG | qRT-PCR |
| qTCTP-F | 5’-CGACCCAACCTTCCTTTACT | qRT-PCR |
| qTCTP-R | 5’-GTTTCCGACACCAACATTAGA | qRT-PCR |
| qOs12g34380-F | 5’-GGAAAACTTTGTCAGAGATGGCA | qRT-PCR |
| qOs12g34380-R | 5’-ATAGCCTTGCAGCCCATTCT | qRT-PCR |
| qOs11g42350-F | 5’-GAACGCTTGTGGTAGACGGA | qRT-PCR |
| qOs11g42350-R | 5’-GCTCTCCATTCCGCTTCAGA | qRT-PCR |
| qOs05g34290-F | 5’-CGCTCGCTTCAAATACCCTC | qRT-PCR |
| qOs05g34290-R | 5’-GCATGAACCCCCTGAGAAGC | qRT-PCR |
| qOs06g01260-F | 5’-ACACAGTGAGTTGCAGAGATGA | qRT-PCR |
| qOs06g01260-R | 5’-ACTAAGCGGGACAGAAGTGC | qRT-PCR |
| qOs04g52900-F | 5’-TTGCACTCTTCGGTGTTTGC | qRT-PCR |
| qOs04g52900-R | 5’-TATGGAGGTGCCAGTAGCGA | qRT-PCR |
| N48Q-F | 5’-TTGATGTGGACATTGGTGCCCAACCATCTGCTGAGGG | Mutagenesis |
| N48Q-R | 5’-TTGGGCACCAATGTCCACATCAATAGCTCCTTGAACGA | Mutagenesis |
| N97Q-F | 5’-TCATGAAGCGCTACATCAAGCAACTCTCCGCCAAGC | Mutagenesis |
| N97Q-R | 5’-TTGCTTGATGTAGCGCTTCATGAAGGTCACAAACTGCT | Mutagenesis |
